# Stability Challenges and Non-Target Effects of Mandelonitrile-Based Sugar Baits for Leishmaniasis Vector Control

**DOI:** 10.3390/insects16111106

**Published:** 2025-10-30

**Authors:** Camila J. Pereira-Pinto, Jean P. S. Costa, Juliana Welbert, João P. D. Simoni, Gabriel S. Thomaz, Ana C. V. Faria, Sergio M. Correa, Bruno Gomes, Fernando A. Genta

**Affiliations:** 1 Laboratory of Insect Biochemistry and Physiology, Oswaldo Cruz Institute—IOC, Oswaldo Cruz Foundation—Fiocruz, Av. Brasil 4365, Pav. 26, S207, Manguinhos, Rio de Janeiro 21040-360, Brazil; kakabiogene@gmail.com (C.J.P.-P.); jeanpaulocosta216@outlook.com (J.P.S.C.); juliana.welbert@hotmail.com (J.W.); joaopdrummond@gmail.com (J.P.D.S.); gsthomaz@edu.unirio.br (G.S.T.); anacaroldovalle@gmail.com (A.C.V.F.); gomesb.phd@gmail.com (B.G.); 2 Laboratory of Insect Biochemistry and Physiology, National Institute of Rural Endemic Diseases—INERU, Oswaldo Cruz Foundation—Fiocruz, Room 3, Estrada da Covanca 66, Tanque, Rio de Janeiro 22735-020, Brazil; 3 Environmental Monitoring Laboratory, Faculty of Technology, State University of Rio de Janeiro—UERJ, Pres. Dutra Highway km 298, Resende 27537-000, Brazil; sergiomc@uerj.br

**Keywords:** leishmaniasis, mandelonitrile, off-target effects, sand flies, sugar baits, vector control

## Abstract

Leishmaniasis is a vector-borne disease of global concern, transmitted by sand flies of the genera *Lutzomyia* and *Phlebotomus*. Attractive toxic sugar baits (ATSBs) have emerged as a promising alternative for vector control, leveraging the sugar-feeding behavior of adult sand flies. Mandelonitrile, a plant-derived compound with potent anti-*Leishmania* activity, has shown promise in reducing parasite load and sand fly longevity. However, key aspects such as bait stability and off-target effects remain poorly understood. In this study, we evaluated the stability of 70% sucrose-based sugar baits supplemented with methylparaben or sodium azide, and quantified mandelonitrile degradation over time using a GC-MS protocol adapted for high-sucrose matrices. We also assessed the physiological impact of mandelonitrile on the non-target model organism *Drosophila melanogaster*. Results demonstrated that methylparaben and sodium azide can stabilize sucrose solutions for up to seven days, although both compounds affected sand fly survival. Mandelonitrile was undetectable after one week, indicating rapid degradation or volatilization. In *D. melanogaster*, mandelonitrile reduced longevity and inhibited oviposition. This is the first report on the stability and off-target effects of mandelonitrile sugar baits, highlighting the need for improved formulations and thorough evaluation of their ecological safety, with the goal of developing transmission-blocking sugar baits for leishmaniasis control.

## Simple Summary

Leishmaniasis is a disease spread by tiny insects called sand flies. Since there is no vaccine and treatment can be difficult, scientists are exploring new ways to stop the insects from spreading the disease. One promising method is using sugar baits—sweet solutions that attract sand flies and contain substances that can kill the parasite or shorten the insect’s life. This study tested sugar baits containing mandelonitrile, a natural compound known to fight the *Leishmania* parasite. We looked at how stable these baits are over time and whether they might harm other insects. We found that mandelonitrile is quickly lost, making the bait less effective after one week. To improve stability, we added preservatives like methylparaben and sodium azide, which helped keep the sugar stable but also affected sand fly survival. To check for unintended effects, we tested the bait on fruit flies (*Drosophila melanogaster*), a common model for studying insect biology. Mandelonitrile reduced their lifespan and egg-laying ability, suggesting potential risks to non-target species. This is the first study to examine both the durability and ecological safety of mandelonitrile sugar baits. The findings highlight the need for better formulations and careful testing to ensure safe and effective control of leishmaniasis.

## 1. Introduction

Leishmaniasis is a group of diseases caused by parasites of the *Leishmania* (Ross, 1903) genus, accounting for approximately 30,000 deaths and over one million cases annually [1]. These parasites are transmitted primarily by sand flies of the genera *Lutzomyia* (Lutz & Neiva, 1912) and *Phlebotomus* (Macquart, 1834) during the bite and blood meal of adult females on a vertebrate host [2].

Due to the lack of vaccines against these parasites, the difficulty of treatment, and the emergence of parasites resistant to the main drugs of choice, the control of insect vectors has gained particular importance [3]. Historically, sand fly control has been achieved with chemical insecticides through residual applications or spatial fumigation, which has raised concerns regarding environmental impacts and the selection of resistant insect populations [4]. Therefore, the development of new, more specific and environmentally friendly control tools is a strategic necessity for Leishmaniasis management programs.

An alternative to traditional chemical control that has been studied in recent years is the use of toxic-attractive sugar baits against sand flies [5]. These consist of a sugar solution containing a chemical or biological insecticide, with or without added attractants such as fruit juices. The solution can be offered to the sand flies by spraying vegetation or on surfaces such as fences or feeding stations [6]. This strategy exploits the feeding habits of adult sand flies, which require sugar sources to meet their daily energy needs. The most common sources in nature are flower nectar, fruit juices, or aphid secretions [7,8]. In addition to being the sole food source for adult males, it has been shown that the sugar diet is essential for female longevity and is also critical for parasite development in the vector [9].

Given this network of ecological interactions, some years ago, Schlein and Jacobson proposed in 1994 that plant-derived compounds could have antiparasitic action within the insect’s gut [10]. This phenomenon has recently been explored in new formulations targeting sand flies, with significant effects on parasite and vector development, as well as on insect longevity and behavior [11,12,13]. Of particular interest is the application of sugar baits containing mandelonitrile, as this compound has a strong anti-*Leishmania* effect against all species tested to date, reducing parasite load and infection prevalence in insects, in addition to decreasing the longevity of males and females [11]. Due to the delivery route of the active ingredient and its multiple effects on both the parasite and the vector, this approach is expected to result in reduced selection pressure for resistance, as well as a reduced environmental impact.

However, relevant topics to the development and application of this tool remain unclear, such as the stability of the baits. Stability of sugar baits is an essential aspect for practical implementation in the field, and in this respect sugar baits targeting sand flies have been poorly studied. One main reason for the existence of this knowledge gap may be the preferred delivery system of spraying vegetation with chemical insecticides, aiming for fast killing and depletion of the vector population. In this context, stability is not a major issue. However, in the context of antiparasitic sugar baits, where continuous delivery may be desirable, it is important to know the active lifespan of the baits to assess the feasibility of their application in the field.

Another critical feature in the adoption of sugar baits in vector control programs is their effects on non-target organisms. Despite the relevance of the subject, as the use of chemical insecticides may harm beneficial insects such as bees and ecological networks, the ecological impacts of sugar baits for sand flies have been scarcely assessed. The complexity of these studies, with the necessity of working with different biological models for toxicity measurements, or extensive taxonomical field work after trials for off-target mapping, is a clear impediment for this line of research.

In this work, we focused on anti-*Leishmania* bait formulation to clarify the points above. Mandelonitrile was chosen as an antiparasitic molecule based on its strong effect on different *Leishmania* species and efficacy in decreasing the vector infection prevalence and parasite load in previous experiments [11]. Besides that, it is a molecule that does not resemble any drug that has been used for the clinical treatment of leishmaniasis, lowering the future possibility of development of cross-resistant parasites in the field. Briefly, we studied the stability of baits in the laboratory, standardizing the addition of preservatives to the sugar solution and following their effects on sand flies of the species *Lutzomyia longipalpis* (Lutz & Neiva, 1912). Furthermore, we characterized the effect of mandelonitrile on a model non-target organism, *Drosophila melanogaster* (Meigen, 1830). The data together point to prospects for the improvement and sustainability of the application of antiparasitic sugar baits for the control of leishmaniasis.

## 2. Materials and Methods

### 2.1. Insects

Sandflies of the species *L. longipalpis*, bred at the Laboratory of Insect Biochemistry and Physiology (Oswaldo Cruz Institute, Rio de Janeiro, Brazil), were used. These insects were collected in the 1990s by Prof. Richard Ward in the city of Jacobina (Bahia State, Brazil) and have been maintained in a closed colony since then according to ref. [14].

*D. melanogaster* strain Canton S was obtained from Prof. Antonio Bernardo Souza de Carvalho (Federal University of Rio de Janeiro, Rio de Janeiro, Brazil) and maintained according to ref. [15]. To produce 500 mL of CC2 medium, the following were used: cornmeal (30 g); agar (4 g); yeast (10 g); sugar (25 g); methylparaben (0.75 g); salt (0.5 g); propionic acid (2 mL); 95% alcohol (3.75 mL) and water (500 mL).

### 2.2. Preparation and Composition of Sugar Baits

The sugar baits used in this work consisted of cotton balls, weighing 30 mg each. Each cotton ball was soaked with 300 µL of sugar solution. Five different formulations were tested independently: (1) 70% (*w*/*v*) sucrose solution (control) (*w*/*v*); (2) 70% (*w*/*v*) sucrose and 0.01% (*w*/*v*) sodium azide solution; (3) 70% (*w*/*v*) sucrose and 0.05% (*w*/*v*) sodium azide solution; (4) 70% (*w*/*v*) sucrose and 0.25% (*w*/*v*) methylparaben solution; (5) 70% (*w*/*v*) sucrose and 0.1% (*w*/*v*) mandelonitrile solution.

Sucrose 70% (*w*/*v*) was used as a control because our *L. longipalpis* colony has been kept on sucrose 70% (*w*/*v*) since its establishment. Besides that, this sugar concentration elicits the optimal response in terms of digestive enzymes [16], resembling the concentration of sucrose in the nectar of several flowering plants [17]. Sodium azide was chosen as an antimicrobial stabilizer for a proof-of-concept approach [18]. Due to the toxicity of sodium azide [18], we also performed tests with methylparaben, a food stabilizer approved by the Food and Drug Administration (USA) and European Medicines Agency (EMA) [19,20,21].

The sucrose solution was prepared with commercial refined sugar and subsequently sterilized in an autoclave. Sodium azide and methylparaben were dissolved in ultrapure water and methyl alcohol, respectively, to produce a 10% (*w*/*v*) solution before combining them with 70% (*w*/*v*) sucrose. Sodium azide and methylparaben were purchased from Sigma-Aldrich (St. Louis, MO, USA) and Dinâmica Química Contemporânea (Indaiatuba, SP, Brazil).

### 2.3. Stability of Sugar Baits

The stability of 70% sucrose was checked in the five sugar bait formulations above. Each experiment was replicated three times, consisting of one homemade cubic fabric cage (15 cm × 15 cm × 15 cm, polyester netting, mesh size 96 × 26, 680 µm aperture) containing 20 sugar baits. The balls were distributed over two Petri dish covers (diameter 10 cm), avoiding contact between them (10 balls per Petri dish, total 6 mL sugar solution per cage). The cages were placed in an incubator oven (BOD) to maintain a temperature of 26 ± 1 °C and an ambient humidity range of 50–70%. During the experiments, the cages were maintained inside closed plastic bags with paper towels that were moistened every 2 days with deionized water. In these conditions, the humidity inside the cages was high (80%), and it was not necessary to replenish the cotton balls with water.

Sucrose degradation was monitored after 0 and 7 consecutive days, where the collected cottons were placed in a falcon tube containing 2 mL of ultrapure water and frozen for later analysis. Glucose was measured in each cotton ball as an indicative of sucrose degradation due to hydrolysis. To detect and quantify glucose in the collected samples, we used the glucose monoreagent kit (BioClin, Belo Horizonte, MG, Brazil) according to manufacturer instructions.

### 2.4. Effect of Bait Exposure to Sandflies

Cages were mounted as described above with each one of the 5 sugar bait formulations inside. After preparing the cages, 10 female and 10 male *L. longipalpis* were placed in the cages, and the cages were kept and monitored as described above. The adult sandflies used in the experiment were between 0 and 2 days old and were not fed previously to encourage them to approach the baits. After 2 and 7 days, the number of dead insects under the different conditions was recorded, and after 7 days the glucose content of each bait was measured as described above. No trapping of sandflies in the sugar solutions was observed in any of the conditions tested.

### 2.5. Quantification and Stability of Mandelonitrile in Sugar Baits

Five independent tests were conducted to detect the presence of mandelonitrile in sugar baits and to assess their stability after one week. In the tests, eight sugar baits were prepared as described above and placed in a Petri dish cover. The sugar solution was composed of 70% (*m*/*v*) sucrose, plus 0.25% (*v*/*v*) methylparaben, and mandelonitrile at a final concentration of 0.1% (*v*/*v*).

The plate containing the sugar baits was placed in a cubic fabric cage as described above. A second cage was prepared identically, with 20 sand flies of the species *L. longipalpis* (10 males and 10 females) being introduced to monitor mortality and the possible effect of their presence on the stability of the mandelonitrile. As in the previous experiments, dead insects were counted, and humidity was maintained by adding water to the paper towels in the cages. Initially, four cotton pieces were collected and stored in 15 mL glass tubes containing 4.7 mL of acetonitrile, which were kept refrigerated for later analysis. The cages were monitored for seven days, after which the remaining four cotton samples were collected and subjected to quantification of the remaining mandelonitrile using gas chromatography-mass spectrometry (GC-MS; Ref. [22]).

Mandelonitrile used as a standard was purchased from Sigma-Aldrich^®^ (St. Louis, MO, USA, code 116025). Standard solutions were prepared at concentrations of 0.75, 1.50, 3.0, 15, 30, and 60 ng/µL, following the protocol described by ref. [22], with adaptations for the analysis of the samples in the present study. Both standards and samples were derivatized by trimethylsilylation with N-methyl-N-trimethylsilyltrifluoroacetamide (MSTFA) (Sigma-Aldrich^®^, St. Louis, MO, USA) to enable detection by GC-MS. Sucrose interference was eliminated by including a sugar precipitation step in the samples with acetonitrile, prior to the derivatization of mandelonitrile, which is fully soluble under the tested conditions.

Briefly, 50 µL of mandelonitrile was transferred to a 2 mL amber vial, to which 100 µL of MSTFA was added. This mixture was homogenized and incubated in an oven at 60 °C for 30 min. After cooling, standard solutions were prepared using acetonitrile as the solvent. The cotton samples previously stored in acetonitrile were vortexed, and a 100 µL aliquot was collected for derivatization as described above. After this step, 200 µL of acetonitrile was added to the MSTFA-derivatized samples.

Coupled GC-MS analyses were performed on a Varian 450GC gas chromatograph, MS220 mass spectrometry detector, and Restek Rxi 5MS chromatographic column, 30 m long, 0.25 mm external diameter, and 0.25 µm stationary phase thickness. The equipment was programmed according to the following conditions: injection temperature 250 °C, injection volume 1.0 µL, sample division in splitless mode, mobile phase flow rate 1.0 mL/min of Helium 5.0 (99.999%), column temperature at 60 °C for 3 min, followed by a heating ramp of 10 °C/min to 230 °C, monitored ions: 76–78, 104–106, and 189–191 *m*/*z* with filament current at 50 µA. The peaks corresponding to mandelonitrile in the analyzed samples were identified by comparing the retention times and mass spectra with those obtained for the compound standard. Mandelonitrile was quantified by comparing the peak areas of known quantities of the standards with the peak areas of the compound observed in the samples.

### 2.6. Exposure of a Non-Target Organism (D. melanogaster) to Mandelonitrile

*Drosophila* was used as a convenient dipteran model for preliminary screening of toxicity against off-target organisms. To observe the potential impact of sugar baits containing mandelonitrile on *D. melanogaster*, a 30 cm acrylic cubic cage was prepared containing a plastic jar with 50 mL of CC2 culture medium and a Petri dish with 0.2 g cotton soaked in 2 mL of a 70% (*w*/*v*) sucrose and 0.1% (*v*/*v*) mandelonitrile solution. A second cage (control) was prepared following the same pattern as the previous cage. However, only 2 mL of the 70% sucrose solution was pipetted onto the cotton. Twenty-five flies (male or female) were added to both cages, and the longevity of the insects was monitored. The number of dead insects under the different conditions was recorded, and the cotton pads containing the solutions and the culture media were changed every 2–3 days. Three independent replicates of this experiment were conducted.

The cages were kept in black plastic bags with moistened paper towels and placed in an incubator oven (BOD) to maintain humidity above 50% and a temperature of 26 ± 1 °C. The paper towels were kept moist at all times to ensure standard experimental conditions.

### 2.7. D. melanogaster Oviposition in Culture Medium

Oviposition preference assays of *D. melanogaster* in culture medium containing mandelonitrile were also performed. Two cubic cages were set up, each containing 25 flies. One plastic pot containing CC2 culture medium containing mandelonitrile at a final concentration of 0.08% (*w*/*w*) and one plastic pot containing only CC2 medium were placed in opposite positions. The cages were kept at room temperature inside black plastic bags with moistened paper towels attached to the outside of the cages.

After 2 days of exposure to the culture media, the pots were collected, covered, and placed in an incubator (BOD) for larvae and pupae counting 5 days after removal from the cages. A total of 6 rounds of this experiment were conducted, with two replicates in each. The locations of the CC2 and CC2 + mandelonitrile pots inside the cages were permuted along the replicas and rounds to avoid positional bias.

### 2.8. Forced Exposure of D. melanogaster to a Diet with Mandelonitrile

To analyze the survival of flies exposed solely to a diet containing mandelonitrile, we set up a cubic cage containing 25 *D. melanogaster* specimens and 1 plastic pot with CC2 culture medium mixed with the study compound at a final concentration of 0.08% (*w*/*w*). A second cage was set up as a control, presenting a similar pattern to the previous cage; however, the culture medium was not treated with mandelonitrile. The flies were exposed to the media for 14 days. Every 2–3 days, the food source (treated and control) was replaced, the dead flies were counted, and the collected pots were stored in a BOD for later larval and pupal counting. To obtain more consistent data, a total of 5 replicates of this experiment were performed.

### 2.9. Statistical Analyses

All statistical analyses were conducted using GraphPad Prism software, version 9.0.2 for Windows, GraphPad Software, Boston, MA, USA, www.graphpad.com. The normality of the distribution of the obtained values was tested using the D’Agostino & Pearson, Anderson-Darling, Shapiro–Wilk, and Kolmogorov–Smirnov tests. To compare glucose concentrations between baits in different groups, the Kruskal–Wallis test was used, followed by Dunn’s multiple comparisons test. Survival curves were compared using the Log-rank (Mantel–Cox) test, allowing for the assessment of statistically significant differences over time. Ratios between the numbers of dead and live insects between different treatments were compared using Fisher’s exact test. Comparisons between mandelonitrile concentrations in baits from different groups, as well as the number of eggs laid after different treatments, were made using the Mann–Whitney test.

## 3. Results

**Figure 1:**
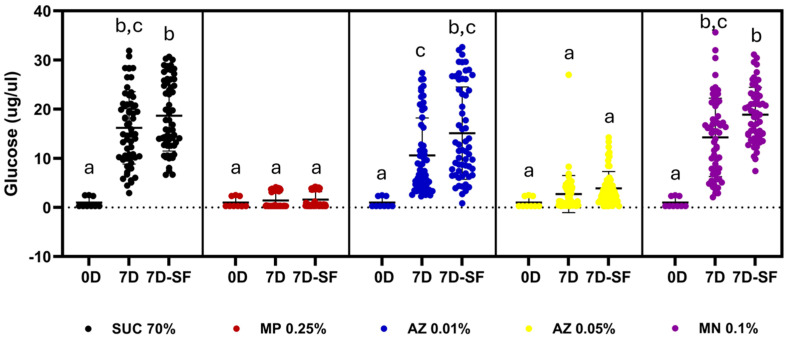
Stability of sucrose (70%, *w*/*v*) in sugar baits after one week of exposure to laboratory conditions in the presence of different stabilizers, compounds, or the presence of adult sand flies. The glucose concentration detected in each bait was used as a marker of degradation by hydrolysis. Traces and error bars correspond to the mean and standard deviation of three independent determinations with 10 baits each (n = 30). 0D—baits before incubation (0 days); 7D—baits after 7 days of incubation; 7D-SF—baits after 7 days of incubation in the presence of sand flies; SUC 70%—baits containing only 70% sucrose (*w*/*v*); MP 0.25%—baits containing 70% sucrose (*w*/*v*) added with 0.25% methylparaben (*w*/*v*); AZ 0.01% (*w*/*v*)—baits containing 70% (*w*/*v*) sucrose added with 0.01% (*w*/*v*) sodium azide; AZ 0.05%—baits containing 70% (*w*/*v*) sucrose added with 0.05% (*w*/*v*) sodium azide; MN 0.1%—baits containing 70% (*w*/*v*) sucrose added with 0.1% (*w*/*v*) mandelonitrile; Different letters indicate significantly different groups (*p* < 0.05, Kruskal–Wallis and Dunn’s test).

The first aspect investigated was the stability of sucrose in the sugar baits used under laboratory conditions. As an indicator of sucrose degradation, glucose generation was measured, as glucose is one of the products of sucrose hydrolysis. Initially, all tested baits had low glucose concentrations (0.98–1.00 µg/µL), with no significant differences between the groups (Figure 1, Dunn’s test, *p* > 0.999, see Supplemental File). The low glucose levels at the beginning of the experiments likely resulted from contamination of the sucrose used to prepare the baits. Exposing sucrose-containing baits to laboratory conditions for 7 days (Figure 1) resulted in a significant increase in glucose concentration, suggesting the occurrence of sucrose hydrolysis. The glucose concentration reached an average concentration 16 times higher than the control (16.2 ± 0.9 µg/µL; Dunn’s test, Z = 5.855, *p* < 0.0001).

After observing that sucrose was not stable under laboratory conditions, we decided to test whether the addition of compounds with known antimicrobial activity, such as methylparaben and sodium azide, could prevent this degradation [18,19,20,21]. In parallel, we also tested the addition of mandelonitrile, also due to its antimicrobial properties [11], although it is not a commonly used stabilizer in industrial or laboratory applications. The addition of 0.25% (*w*/*v*) methylparaben resulted in the stabilization of the sucrose solution, observing a glucose concentration after 7 days (1.4 ± 0.2 µg/µL) not significantly different from the initial concentration (0.9 ± 0.3 µg/µL; Dunn’s test Z = 0.7489, *p* > 0.9999; Figure 1 and Supplementary File). The results observed after the addition of sodium azide differed in relation to the dose used. While a concentration of 0.01% (*w*/*v*) NaN3 was not able to stabilize the sugar solution, resulting in a glucose concentration after 7 days of incubation (10.5 ± 0.9 µg/µL) significantly higher (10.8×) than the initial concentration (0.9 ± 0.3 µg/µL, Dunn’s test Z = 4.422, *p* = 0.001), the same compound at a concentration of 0.05% (*w*/*v*) resulted in glucose concentrations after 7 days of incubation similar to those of the controls (2.7 ± 0.4 µg/µL, Dunn’s test Z = 1.689, *p* > 0.9999; Figure 1 and Supplementary File). Regarding mandelonitrile, at a concentration of 0.1% (*w*/*v*), no stabilization of the sugar solution was observed either. After 7 days of incubation, the glucose concentration (14 ± 1 µg/µL) was 14.5 times higher compared to the initial concentration (0.9 ± 0.3 µg/µL, Dunn’s test Z = 5.42, *p* < 0.0001; Figure 1 and Supplementary File).

After observing that sucrose was not stable under laboratory conditions, despite the high concentration tested in the baits (70%, *w*/*v*), we decided to investigate the effect that exposing the baits to adult sand flies could have on this phenomenon (Figure 1). Although the feeding behavior of sand flies during the ingestion of sugar solutions is little explored in experimental studies, it is quite likely that the baits are inoculated with insect saliva, which may include both microorganisms and enzymes or antimicrobial agents [23,24], so that insect feeding could alter the behavior of sugar solutions after 7 days of laboratory exposure.

Although we observed a small increase of 15% in the glucose concentration in the baits exposed to insects for 7 days (18.6 ± 0.9 µg/µL) when compared to the controls incubated without insects (16.2 ± 0.9 µg/µL), no statistically significant difference was observed between the two groups (Dunn’s test Z = 1.013, *p* > 0.9999; Figure 1 and Supplementary File). A similar response was observed in the tests with methylparaben 0.25% (*w*/*v*), where exposure to insects resulted in an 11% increase in glucose concentration (1.5 ± 0.2 µg/µL), compared to controls incubated without insects (1.4 ± 0.2 µg/µL), which was not statistically significant (Dunn’s test, Z = 0.768, *p* > 0.9999; Figure 1 and Supplementary File). In experiments performed with 0.01% (*w*/*v*) azide, the presence of insects resulted in a glucose concentration (15 ± 1 µg/µL) 42% higher than that observed in control baits after 7 days of incubation (10.5 ± 0.9 µg/µL), but the difference was not statistically significant (Dunn’s test Z = 2.071, *p* > 0.9999; Figure 1 and Supplementary File). The exposure of baits containing 0.05% (*w*/*v*) azide to sand flies for 7 days resulted in a glucose concentration (3.8 ± 0.4 µg/µL) also 42% higher than that observed in controls incubated for 7 days without the insects (2.7 µg/µL), an elevation that was also not significant (Dunn’s test Z = 0.97, *p* > 0.9999, Figure 1 and Supplementary File). Finally, no significant effects were observed when insects were exposed to sucrose solutions containing 0.1% (*w*/*v*) mandelonitrile for 7 days, despite a 32% increase in glucose concentration, from 14 ± 1 µg/µL to 18.8 ± 0.7 µg/µL (Dunn’s test Z = 2.159, *p* > 0.9999; Figure 1 and Supplementary File).

**Figure 2:**
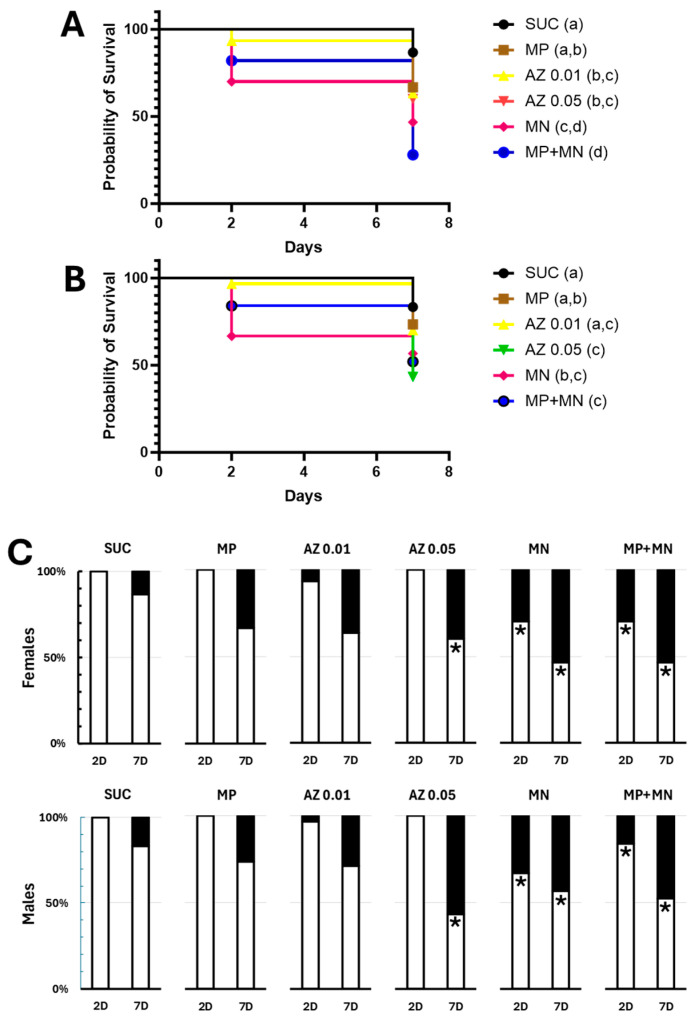
Effect of sugar baits containing different compounds on the survival of sand flies. (**A**) Survival curves of *L. longipalpis* females exposed to the baits for 7 days. (**B**) Survival curves of *L. longipalpis* males exposed to the baits for 7 days. (**C**) Percentage of live and dead sand flies after 2 or 7 days of exposure. Each cage contained 10 males and 10 females. SUC—baits containing only 70% (*w*/*v*) sucrose; MP—baits containing 70% (*w*/*v*) sucrose added with 0.25% (*w*/*v*) methylparaben; AZ 0.01—baits containing 70% (*w*/*v*) sucrose added with 0.01% (*w*/*v*) sodium azide; AZ 0.05—baits containing 70% (*w*/*v*) sucrose added with 0.05% (*w*/*v*) sodium azide; MN—baits containing 70% (*w*/*v*) sucrose added with 0.1% (*w*/*v*) mandelonitrile; MP+MN—baits containing 70% (*w*/*v*) sucrose added with 0.25% (*w*/*v*) methylparaben and 0.1% (*w*/*v*) mandelonitrile; 2D—2 days of exposure; 7D—7 days of exposure. White bars correspond to the percentage of live insects, and black bars correspond to the percentage of dead insects. In (**A**,**B**), different letters (a–d) indicate significantly different groups (*p* < 0.05, Log-rank test). In (**C**), asterisks indicate significantly different ratios of dead to live insects when compared to the SUC group, with the same incubation time, 2 or 7 days (*p* < 0.05, Fisher’s exact test).

A previously described effect of sugar baits containing mandelonitrile is a reduction in the longevity of sand flies, especially males [11]. Therefore, we decided to verify whether the conditions tested were resulting in significant mortalities over the 7 days of experimentation (Figure 2A–C and Supplementary File). The 70% (*w*/*v*) sucrose control solution resulted in mortality levels considered low, below 20%, being 13% for females and 17% for males (Figure 2A–C and Supplementary File), which is compatible with the survival curves of *L. longipalpis* previously described. Exposure to 0.25% methylparaben resulted in slightly higher mortality levels, especially after 7 days (33% for females and 27% for males, Figure 2C and Supplementary File), but not significantly different from those observed in controls (Fisher’s exact test, *p* = 0.12 and *p* = 0.53, respectively). Also, the temporal patterns of survival were also not significantly altered (Log-rank test, χ^2^ = 3.298, *p* = 0.0694 and χ^2^ = 0.8691, *p* = 0.3512, respectively) (Figure 2A,B and Supplementary File).

Exposure to 0.01% (*w*/*v*) azide resulted in slightly higher mortalities after 7 days (37% for females and 29% for males, Figure 2C), but not significantly different from controls fed only sucrose (Fisher’s exact test, *p* = 0.0716 and *p* = 0.3627, respectively). However, a significant change in the survival pattern of females was observed (Log-rank test, χ^2^ = 4.541, *p* = 0.0331, Figure 2A), an effect that was not observed in males (Log-rank test, χ^2^ = 1.575, *p* = 0.2095, Figure 2B). Exposure to 0.05% (*w*/*v*) azide resulted in cumulative mortalities after 7 days of 40% in females and 57% in males, significantly higher than those observed in the control group (Fisher’s exact test, *p* = 0.039 and *p* = 0.0028, respectively; Figure 2C and Supplementary File). Also, we observed altered longevity patterns for both sexes (Log-rank test, χ^2^ = 5.364, *p* = 0.0206 and χ^2^ = 10.16, *p* = 0.0014, respectively, Figure 2A,B). It is interesting to note, however, that the longevity patterns observed in the two azide concentrations did not differ significantly from each other (Log rank test, χ^2^ = 0.005, *p* = 0.94 and χ^2^ = 3.667, *p* = 0.0555, respectively, for females and males; Figure 2A,B and Supplementary File).

Mandelonitrile exposure resulted in 7-day mortalities of 53% for females and 43% for males, significantly higher than those of controls (Fisher’s exact test, *p* = 0.0022 and *p* = 0.047, respectively; Figure 2C and Supplementary File). Furthermore, significantly altered longevity patterns were observed for both sexes (Log rank test, χ^2^ = 11.92, *p* = 0.0006 and χ^2^ = 6.121, *p* = 0.0134, respectively, for females and males; Figure 2A,B and Supplementary File). For baits containing 0.25% (*w*/*v*) methylparaben and 0.01% (*w*/*v*) mandelonitrile, mortality rates of 72% for females and 48% for males were observed, significantly higher than those of the control (Fisher’s exact test, *p* < 0.0001 and *p* = 0.0076, respectively; Figure 2C and Supplementary File). Longevity patterns significantly different from those of the controls were also observed for both sexes (Log rank test, χ^2^ = 25.02, *p* < 0.0001 and χ^2^ = 8.476, *p* = 0.0036, respectively, for females and males; Figure 2A,B and Supplementary File).

**Figure 3:**
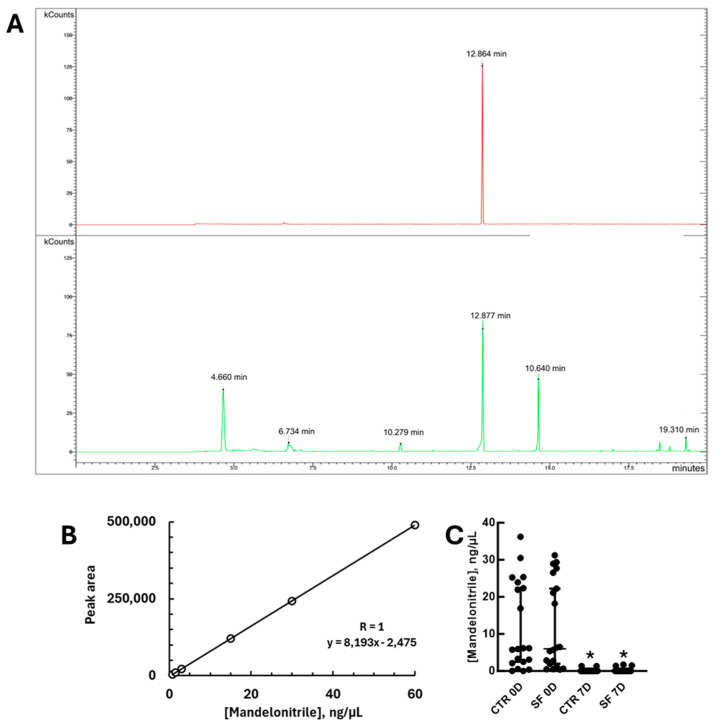
Quantification of mandelonitrile by gas chromatography. (**A**) Chromatograms illustrating the detection profiles of samples after derivatization with MSTFA and GC analysis. Upper panel—mandelonitrile standard; Lower panel—sample from a freshly prepared sugar bait. kCounts—intensity of relative detection. (**B**) Standard curve of mandelonitrile of samples subjected to the acetonitrile precipitation procedure followed by derivatization with MSTFA. (**C**) Quantification of mandelonitrile in sugar baits before and after incubation for 7 days, in the absence or presence of sandflies in the cages. CTR 0D—baits before incubation (0 days) in the absence of sandflies; SF 0D—baits before incubation (0 days) in the presence of sandflies; CTR 7D—baits after 7 days of incubation in the absence of sandflies; SF 7D—baits after 7 days of incubation in the presence of sandflies. In (**C**), asterisks indicate significantly different concentrations when compared to baits before incubation, in the absence or presence of sand flies (*p* < 0.05, Mann–Whitney test).

The next aspect we decided to study was the stability of mandelonitrile under the conditions tested. However, specific and quantitative detection techniques for mandelonitrile are rarely described in the literature. Among the previously described methods—HPLC, GC-MS, and GC—we chose gas chromatography because of its speed, simplicity, high sensitivity, direct detection of the compound, and specificity. However, application of the protocol available in the literature [22] to sugar solutions containing mandelonitrile did not yield detectable signals, suggesting that the presence of sucrose interferes with the derivatization protocol and application in gas chromatography. Therefore, we included a sample precipitation step with acetonitrile, which resulted in the complete removal of sucrose and quantification of mandelonitrile at levels comparable to those in the literature. Under these conditions, after derivatization with MSTFA, mandelonitrile was detected as an ionic product with a retention time of 12 min and an *m*/*z* of 190, which is consistent with previous reports (Figure 3A; Ref. [21]). While ref. [22] report detections in the range of 5 to 500 ng/µL, we observed good linearity between 0.75 and 60 ng/µL from sugar solutions (Figure 3B). This corresponded to 0.075–6% of the concentration in baits containing 0.1% (*w*/*v*) of this compound.

After standardizing the detection, we applied the technique to measure mandelonitrile levels in sugar baits after 7 days of incubation under laboratory conditions. This was performed in the absence or presence of sand flies in the cages (Figure 3C). Mandelonitrile was detectable in all control baits before incubation, although with significant variation between samples. However, after 7 days of exposure, the amount of mandelonitrile in the baits was significantly lower or even undetectable. The same result was observed regardless of the presence or absence of sand flies in the cages (Figure 3C; Mann–Whitney test, U = 26, *p* < 0.0001 and U = 15, *p* < 0.0001, respectively).

**Figure 4:**
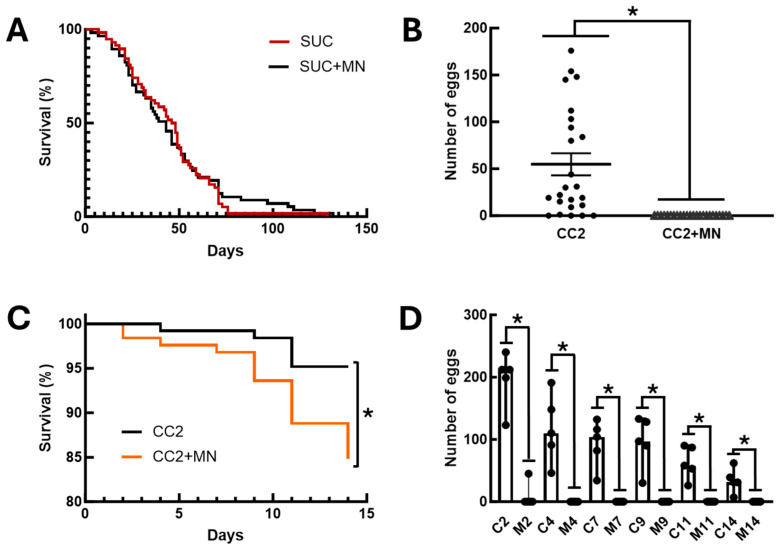
Effects of mandelonitrile on the non-target organism, the fly *D. melanogaster*. (**A**) Survival curves of adults (male and female) exposed to sugar baits placed inside the cage. SUC—baits with 70% (*w*/*v*) sucrose solution; SUC+MN—70% (*w*/*v*) sucrose baits containing 0.1% (*w*/*v*) mandelonitrile. (**B**) Number of eggs laid by adult *D. melanogaster* females in a free-choice experiment between two oviposition and larval development media. CC2—control nutrient medium CC2 for maintaining *D. melanogaster* larvae; CC2+MN—CC2 medium containing 0.08% (*w*/*w*) mandelonitrile. (**C**) Survival curves of adult *D. melanogaster* females during forced exposure to CC2 or CC2+MN oviposition media. See Methods for composition. (**D**) Number of eggs laid over time in the experiment of forced exposure to CC2 or CC2+MN media. C2, C4, C7, C9, C11, and C14—number of eggs laid after 2, 4, 7, 9, 11, and 14 days of exposure to CC2 medium, respectively; M2, M4, M7, M9, M11, and M14—number of eggs laid after 2, 4, 7, 9, 11, and 14 days of exposure to CC2+MN medium, respectively. In (**B**), the asterisk indicates significantly different numbers of eggs in the comparison between groups (*p* < 0.05, Mann–Whitney test). In (**C**), the asterisk indicates significantly different survival curves (*p* < 0.05, Log-rank test). In (**D**), the asterisk indicates significantly different numbers of eggs in the comparison between the control (**C**) and mandelonitrile (M) groups, with the same incubation time, 2 to 14 days (*p* < 0.05, Mann–Whitney test).

An additional aspect investigated was the impact of sugar baits on the physiology of a non-target organism. To this end, we investigated the effect of sugar baits and diets containing mandelonitrile on the fly *D. melanogaster*. Initially, we assessed the effect of exposing adults of this species to these sugar baits. No differences were observed between the longevity of flies exposed to baits containing 0.1% (*w*/*v*) mandelonitrile and insects fed only 70% sucrose (Figure 4A, Log-rank test, χ^2^ = 0.1286; *p* = 0.7199). After this observation, we decided to investigate whether mandelonitrile would have any effect on oviposition or development of immature flies. Adult flies showed a significant preference for oviposition on the CC2 control diet. No eggs were observed on the diet containing mandelonitrile (Figure 4B, Mann–Whitney test, U = 48, *p* < 0.0001).

Because no eggs were observed in the diet containing mandelonitrile in the choice assays, forced oviposition experiments were performed on these two diets to observe the effect of mandelonitrile on larval development. In this new experimental setup, we observed significantly higher mortality in the groups of flies presented with CC2 medium containing mandelonitrile, when compared to flies presented with the CC2 control medium (Figure 4C, Log-rank test, χ^2^ = 7.472, *p* = 0.0063). Furthermore, throughout this experiment we observed that flies avoided oviposition in the CC2 medium containing mandelonitrile. Significantly higher numbers of eggs were observed in the CC2 control group, when compared to the experimental group in which mandelonitrile was added to the same diet (Figure 4D, Mann–Whitney test, U = 0, *p* = 0.0079).

## 4. Discussion

In a previous work, it was shown that sugar baits containing mandelonitrile, a molecule with strong anti-*Leishmania* activity, were able to reduce the longevity, prevalence of infection and vectorial capacity of the sandfly *L. longipalpis* [11]. However, important aspects for the future use of these baits in the field, such as stability or effects on off-target species, have not been characterized yet. This study demonstrated that sugar baits, even using a high sucrose concentration such as 70% (*w*/*v*), are not stable for one week under laboratory conditions. However, degradation could be controlled using antimicrobial agents such as methylparaben 0.25% (*w*/*v*) and sodium azide 0.05% (*w*/*v*). Sodium azide, at a lower concentration (0.01% *w*/*v*), and mandelonitrile (0.01%), despite having antimicrobial properties, were not able to protect the sucrose in the baits from hydrolysis over one week. In most of the different initiatives carried out to control sand flies through sugar baits, stabilizers were not added to the sugar solution. In general, it was only a mixture containing sucrose, a dye, and one or more active ingredients, usually insecticides [25,26,27,28,29,30]. Some exceptions are the works in references [5,6,31], where a commercial stabilizer (BaitStabH; Westham Ltd., Tel Aviv, Israel) was added. However, in none of these initiatives was the stability of the sugar solution monitored. In most studies, the sugar solution is sprayed onto vegetation or cotton or fabric supports, with the aqueous solvent evaporating after administration, meaning the compounds do not remain in solution. This approach cannot be compared with the approach used here. An exception is the work [30], where the stability of a bacterial suspension in sucrose solution was monitored over several days. However, that study did not verify the stability of the sugar in solution, only the viability of the microorganisms, which was its focus. Thus, we verified for the first time the degradation of sugar in sugar solutions offered to sand flies. In addition, we demonstrated its stabilization using two low-cost and easily obtainable compounds, methylparaben and sodium azide. Considering the possible environmental effects associated with sandfly control with sugar solutions, it would be important to extend the studies to more environmentally friendly stabilizers.

Beyond the shelf stability, for practical applications it is important to consider changes in the composition of sugar baits due to their interactions with insects, such as contacting or feeding. Interestingly, although we did not observe statistically significant effects, exposure to sandflies consistently increased glucose levels in the baits incubated for 7 days. This phenomenon may be related to the presence of microorganisms or hydrolytic enzymes in the insects’ saliva. The presence of microorganisms in the saliva of sand flies is a well-known phenomenon, and is important for the transmission of *Leishmania* to the vertebrate host during food ingestion [23,32,33]. Furthermore, the production of digestive sucrases related to the digestion of sugar diets, with impacts on *Leishmania* infection, has also been reported in *L. longipalpis* [16,34]. It is important to highlight that, although sucrase activity in the midgut is elevated and induced by sugar feeding, there are no reports of this activity in sand fly saliva. However, it is important to highlight that transcripts identified as amylases have been described in the salivary glands of *Phlebotomus papatasi* (Scopoli, 1786) [24]. Although amylases generally do not have significant activity on sucrose, they are enzymes that belong to family 13 of glycoside hydrolases, which also includes sucrases and maltases [35]. Therefore, it is possible that sand fly saliva contains sucrase, since the specificity of the enzymes encoded by salivary transcripts is not known. Considering the intricated interactions between sand flies, gut or environmental microbes, and *Leishmania*, it would be interesting in the future to understand whether the presence of glucose, putatively caused by microbial hydrolysis of sucrose, is correlated with changes in the microbiota of the sugar baits, resulting in changes in sand fly feeding behavior or *Leishmania* transmission.

An important limitation to consider in this work is that our study was limited to a one-week proof-of-concept. It would be important to verify the stabilization of the solutions over longer periods, as some control strategies rely on active ingredient half-lives ranging from a few weeks to months, such as organophosphates and neonicotinoids. However, it is important to note that some insecticides, which are well-suited for integrated management strategies, have short half-lives of a few days, such as spinosad and azadirachtin. This stabilization strategy still needs to be validated for different active ingredients.

Assuming the interaction of sand flies with a stable formulation of sugar baits in the field over weeks, a relevant aspect when proposing anti-*Leishmania* baits is the mortality of the insects. Targeting the development of the parasite in the vector, rather than the vector itself, has the advantage of theoretically resulting in less pressure for the selection of insecticide-resistant insects, in addition to a reduced environmental impact. However, the inclusion of the preservative sodium azide in the sugar solutions resulted in significantly higher mortality rates in both sexes. It is important to note that methylparaben did not result in significant mortality when used alone, nor did it increase mortality caused by mandelonitrile, making it an interesting option in this context.

Indeed, a significant reduction in longevity was already reported in insects fed mandelonitrile, with the effect being more pronounced in males than in females [11]. After one week of exposure, mortality was approximately 70% in males and 40% in females. These values differ from those observed in our experiments, where we observed mortality rates of approximately 50% in both males and females. It is possible that variations in the microenvironment are related to these differences, especially changes in temperature and humidity in the experimental cages. It is important to consider the extrinsic incubation time of *Leishmania* in *L. longipalpis*, approximately 7 to 10 days [8], which suggests that longer exposures to baits may be necessary to see effects on parasite establishment and transmission. In any case, due to the toxic effect of mandelonitrile on insects, this strategy tends to combine the sum of the effects on the vector and the parasite on vector competence.

After standardizing glucose quantification in the baits and stabilizing them for at least a week with methylparaben and azide, we decided to investigate whether mandelonitrile underwent a similar degradation process. Importantly, mandelonitrile was detectable and quantified in the sugar baits in the experiments before incubation. However, after 7 days, the mandelonitrile signal was completely lost. Because the stabilizer methylparaben was used in these mandelonitrile stability experiments, it is unlikely that it was degraded by microorganisms, being probably lost by volatilization. In any case, it is interesting to note that, since mandelonitrile was not maintained in the baits throughout the experiment, the effects observed in the sandflies likely resulted from exposure during the first few days of the experiment which may explain the difference between mortality observed in these experiments and those reported by ref. [11]. An interesting approach to solving this problem may be the micro- or nano-encapsulation of mandelonitrile in liposomal carriers, for example.

The last aspect studied was the possible off-target effects of sugar baits containing mandelonitrile. In these studies, we used *D. melanogaster* as a model organism, which proved interesting because it is an insect of the order Diptera, the same as sandflies. However, despite the importance of *D. melanogaster* as a biomedical model, its ecological relevance is limited, so our results must be considered as a preliminary screening rather than a definitive risk assessment. In general, we observed that adult insects were not affected by the sugar baits, but they did avoid ovipositing on diets containing mandelonitrile. We also observed that forced exposure to a larval diet containing mandelonitrile reduces adult longevity, and results in severe inhibition of oviposition. An interesting perspective for future studies will be testing whether mandelonitrile also inhibits the oviposition of sandflies, which could be a novel strategy for the control of these vectors.

The environmental impact of using sugar baits to control sand flies is a nascent topic in the literature. One exception is the study in ref. [5], which observed the impact of spraying vegetation with a toxic sugar solution to control *P. papatasi*. Approximately 8% of marked individuals were observed on insects of different orders such as Coleptera, Lepidoptera, and Hymenoptera collected after 24 h of environmental exposure. No significant effects were reported on the order Diptera, which may be related to the behavioral selectivity of these insects, as observed in our studies. In any case, the observed effect suggests that the impact of baits on other Diptera can be reduced due to deterrence effects. This can certainly be explored in the future in other biotechnological contexts. In general, it is important to highlight that numerous practical aspects of developing sugar bait applications for sand fly control require future investigation. Our studies suggest that studies on bait stability, as well as their effects on insect longevity and other organisms, are crucial to paving the way for the sustainability of this strategy. Furthermore, the development of analytical strategies for chemical characterization of the added compounds is crucial for the viability of these lines of research and experimental work.

## 5. Conclusions

Sugar-based antiparasitic baits containing mandelonitrile can be stabilized by adding methylparaben or azide. However, these compounds can have significant effects on the longevity of sand flies, as well as on other insect species. However, developing techniques to monitor the presence of compounds with antiparasitic activity in concentrated sucrose solutions, as well as their stabilization, is essential for developing this leishmaniasis management strategy.

## Data Availability

The original contributions presented in this study are included in the article/Supplementary Materials. Further inquiries can be directed to the corresponding author.

## Supplementary Materials

The following supporting information can be downloaded at: https://www.mdpi.com/article/10.3390/insects16111106/s1, Supplementary File: Raw data and statistical analysis corresponding to Results in Figure 1, Figure 2, Figure 3 and Figure 4.

## References

[1] World Health Organization (WHO) (2023). The Global Epidemiology of Leishmaniasis.

[2] Tom A., Kumar N.P., Kumar A., Saini P. (2023). Interactions between *Leishmania* parasite and sandfly: A review. Parasitol. Res..

[3] Ayala A., Llanes A., Lleonart R., Restrepo C.M. (2024). Advances in *Leishmania* vaccines: Current development and future prospects. Pathogens.

[4] Balaska S., Fotakis E.A., Chaskopoulou A., Vontas J. (2021). Chemical control and insecticide resistance status of sand fly vectors worldwide. PLoS Neglected Trop. Dis..

[5] Qualls W.A., Müller G.C., Khallaayoune K., Revay E.E., Zhioua E., Kravchenko V.D., Arheart K.L., Xue R.D., Schlein Y., Hausmann A. (2015). Control of sand flies with attractive toxic sugar baits (ATSB) and potential impact on non-target organisms in Morocco. Parasites Vectors.

[6] Müller G.C., Schlein Y. (2011). Different methods of using attractive sugar baits (ATSB) for the control of *Phlebotomus papatasi*. J. Vector Ecol..

[7] Añez N., Lugo A., Loaiza A., Nieves E., Orozco J. (1994). Sugars in the alimentary canal of *Lutzomyia youngi* (Diptera: Phlebotominae). Med. Vet. Entomol..

[8] Ready P.D. (2013). Biology of phlebotomine sand flies as vectors of disease agents. Annu. Rev. Entomol..

[9] Moraes C.S., Aguiar-Martins K., Costa S.G., Bates P.A., Dillon R.J., Genta F.A. (2018). Second blood meal by female *Lutzomyia longipalpis*: Enhancement by oviposition and its effects on digestion, longevity, and *Leishmania* infection. Biomed Res. Int..

[10] Schlein Y., Jacobson R.L. (1994). Mortality of *Leishmania major* in *Phlebotomus papatasi* caused by plant feeding of the sand flies. Am. J. Trop. Med. Hyg..

[11] Ferreira T.N., Pita-Pereira D., Costa S.G., Brazil R.P., Moraes C.S., Díaz-Albiter H.M., Genta F.A. (2018). Transmission blocking sugar baits for the control of *Leishmania* development inside sand flies using environmentally friendly beta-glycosides and their aglycones. Parasites Vectors.

[12] Ferreira T.N., Brazil R.P., McDowell M.A., Cunha-Júnior E.F., Costa P.R.R., Netto C.D., Santos E.C.T., Genta F.A. (2022). Effects of anti-*Leishmania* compounds in the behavior of the sand fly vector *Lutzomyia longipalpis*. Pest Manag. Sci..

[13] Ferreira T.N., Latgé S.G.C., Ramos T.D., Demidoff F.C., Cunha-Júnior E.F., Costa P.R.R., de Souza M.V.N., Brandão Gomes C.R., Dillon V.M., Netto C.D. (2025). Evaluation of sugar meal administered anti-*Leishmania* compounds on the vectorial capacity of the vector, *Lutzomyia longipalpis*. PLoS ONE.

[14] Moraes C.S., Lucena S.A., Moreira B.H., Brazil R.P., Gontijo N.F., Genta F.A. (2012). Relationship between digestive enzymes and food habit of *Lutzomyia longipalpis* (Diptera: Psychodidae) larvae: Characterization of carbohydrases and digestion of microorganisms. J. Insect Physiol..

[15] Ashburner M., Roote J., Sullivan W., Ashburner M., Hawley R.S. (2000). Laboratory culture of *Drosophila*. Drosophila Protocols.

[16] da Costa S.G., Bates P., Dillon R., Genta F.A. (2019). Characterization of α-glucosidases from *Lutzomyia longipalpis* reveals independent hydrolysis systems for plant or blood sugars. Front. Physiol..

[17] Bordin D.M., Latgé S.G.C., Pyke G., Kalman J., Doble P., Genta F.A., Blanes L. (2020). A simple approach to analyze sugar nectar composition in flowers using capillary electrophoresis and enzymatic assays. J. Braz. Chem. Soc..

[18] National Institute for Occupational Safety and Health (NIOSH) (2011). Sodium Azide: Systemic Agent. Centers for Disease Control and Prevention. https://www.cdc.gov/niosh/ershdb/emergencyresponsecard_29750027.html.

[19] Haldar S., Mukherjee S., Dey A. (2025). Never say yes to parabens? A review on paraben compounds in potential human exposure risks. J. Basic Appl. Zool..

[20] ChemicalBook (2025). Methylparaben—Safety Data Sheet.

[21] European Medicines Agency (EMA) (2015). Reflection Paper on the Use of Methyl- and Propylparaben as Excipients in Human Medicinal Products for Oral Use. EMA/CHMP/SWP/272921/2012. https://www.ema.europa.eu/en/documents/scientific-guideline/reflection-paper-use-methyl-and-propylparaben-excipients-human-medicinal-products-oral-use_en.pdf.

[22] Arnaiz A., Vallejo-García J.L., Vallejos S., Diaz I. (2023). Isolation and quantification of mandelonitrile from *Arabidopsis thaliana* using gas chromatography/mass spectrometry. Bio Protoc..

[23] Maleki-Ravasan N., Ghafari S.M., Najafzadeh N., Karimian F., Darzi F., Davoudian R., Farshbaf Pourabad R., Parvizi P. (2024). Characterization of bacteria expectorated during forced salivation of the *Phlebotomus papatasi*: A neglected component of sand fly infectious inoculums. PLoS Neglected Trop. Dis..

[24] Abdeladhim M., Jochim R.C., Ben Ahmed M., Zhioua E., Chelbi I., Cherni S., Louzir H., Ribeiro J.M., Valenzuela J.G. (2012). Updating the salivary gland transcriptome of *Phlebotomus papatasi* (Tunisian strain): The search for sand fly-secreted immunogenic proteins for humans. PLoS ONE.

[25] Schlein Y. (1987). Marking of *Phlebotomus papatasi* (Diptera: Psychodidae) by feeding on sprayed, coloured sugar bait: A possible means for behavioural and control studies. Trans. R. Soc. Trop. Med. Hyg..

[26] Robert L.L., Perich M.J., Schlein Y., Jacobson R.L., Wirtz R.A., Lawyer P.G., Githure G.I. (1997). Phlebotomine sand fly control using bait-fed adults to carry the larvicide *Bacillus sphaericus* to the larval habitat. J. Am. Mosq. Control Assoc..

[27] Mascari T.M., Foil L.D. (2010). Laboratory evaluation of insecticide-treated sugar baits for control of phlebotomine sand flies (Diptera: Psychodidae). J. Am. Mosq. Control Assoc..

[28] Saghafipour A., Vatandoost H., Zahraei-Ramazani A.R., Yaghoobi-Ershadi M.R., Rassi Y., Shirzadi M.R., Akhavan A.A. (2016). Bioassay evaluation of residual activity of attractive toxic sugar-treated barrier fence in the control of *Phlebotomus papatasi* (Diptera: Psychodidae). J. Vector Borne Dis..

[29] McDermott E.G., Morris E.K., Garver L.S. (2019). Sodium ascorbate as a potential toxicant in attractive sugar baits for control of adult mosquitoes (Diptera: Culicidae) and sand flies (Diptera: Psychodidae). J. Med. Entomol..

[30] Ghassemi M., Akhavan A.A., Ramezani A.Z., Yakhchali B., Zarean M.R., Jafari R., Oshaghi M.A. (2024). Assessing survival of transgenic bacteria, *Serratia* AS1 and *Enterobacter cloacae*, in sugar bait, white saxaul plant (*Haloxylon persicum*) and rodent burrow’s soil: A contained-field study for paratransgenesis approach. J. Arthropod-Borne Dis..

[31] Schlein Y., Müller G.C. (2010). Experimental control of *Phlebotomus papatasi* by spraying attractive toxic sugar bait (ATSB) on vegetation. Trans. R. Soc. Trop. Med. Hyg..

[32] Tabbabi A., Mizushima D., Yamamoto D.S., Kato H. (2022). Sand Flies and Their Microbiota. Parasitologia.

[33] Dey R., Joshi A.B., Oliveira F., Pereira L., Guimarães-Costa A.B., Serafim T.D., de Castro W., Coutinho-Abreu I.V., Bhattacharya P., Townsend S. (2018). Gut Microbes Egested during Bites of Infected Sand Flies Augment Severity of Leishmaniasis via Inflammasome-Derived IL-1β. Cell Host Microbe.

[34] da Costa-Latgé S.G., Bates P., Dillon R., Genta F.A. (2021). Characterization of Glycoside Hydrolase Families 13 and 31 reveals expansion and diversification of α-amylase genes in the phlebotomine *Lutzomyia longipalpis* and modulation of sandfly glycosidase activities by *Leishmania* infection. Front. Physiol..

[35] Drula E., Garron M.L., Dogan S., Lombard V., Henrissat B., Terrapon N. (2022). The carbohydrate-active enzyme database: Functions and literature. Nucleic Acids Res..

