# Supplementary material for: Stability Challenges and Non-Target Effects of Mandelonitrile-Based Sugar Baits for Leishmaniasis Vector Control
Source: Insects. 2025 Oct 30;16(11):1106. doi: 10.3390/insects16111106 (PMC12653148; doi:10.3390/insects16111106)
Supplement: Supplementary file 1 [file insects-16-01106-s001.zip › insects-3872388-supplementary.pdf]

Statistical analysis of data of Figure 1. Stability of sucrose (70%, w/v) in sugar baits after one week of exposure to laboratory conditions in the presence of different stabilizers, compounds, or the presence of adult sand flies.

Kluskal -Wallis (Dunn's post-hoc)

	mean	SEM	Z | p	SUC 0D	SUC 7D	SUC SF	MP 0D	MP 7D	MP SF	AZ 0.01% 0D	AZ 0.01% 7D	AZ 0.01% SF	AZ 0.05% 0D	AZ 0.05%7D	AZ 0.05% SF	MN 0D	MN 7D	MN SF
SUC 0D	1,001	0,3617	SUC 0D		<0,0001	<0,0001	>0,9999	>0,9999	>0,9999	>0,9999	0,001	<0,0001	>0,9999	>0,9999	>0,9999	>0,9999	<0,0001	<0,0001
SUC 7D	16,2	0,9518	SUC 7D	5,855		>0,9999	<0,0001	<0,0001	<0,0001	<0,0001	0,5136	>0,9999	<0,0001	<0,0001	<0,0001	<0,0001	>0,9999	>0,9999
SUC SF	18,66	0,9255	SUC 7D SF	6,373	1,013		<0,0001	<0,0001	<0,0001	<0,0001	0,0136	>0,9999	<0,0001	<0,0001	<0,0001	<0,0001	>0,9999	>0,9999
MP 0D	0,9778	0,3543	MP 0D	0,1069	5,996	6,514		>0,9999	>0,9999	>0,9999	0,0005	<0,0001	>0,9999	>0,9999	>0,9999	>0,9999	<0,0001	<0,0001
MP 7D	1,418	0,2042	MP 7D	0,6079	10,27	11,29	0,7489		>0,9999	>0,9999	<0,0001	<0,0001	>0,9999	>0,9999	0,2004	>0,9999	<0,0001	<0,0001
MP SF	1,579	0,2101	MP 7D SF	1	9,505	10,52	1,141	0,768		>0,9999	<0,0001	<0,0001	>0,9999	>0,9999	>0,9999	>0,9999	<0,0001	<0,0001
AZ 0.01% 0D	0,9811	0,3509	AZ 0.01% 0D	0,0032	5,859	6,377	0,1037	0,6121	1,004		0,001	<0,0001	>0,9999	>0,9999	>0,9999	>0,9999	<0,0001	<0,0001
AZ 0.01% 7D	10,59	0,9832	AZ 0.01% 7D	4,418	2,814	3,828	4,559	7,459	6,691	4,422		>0,9999	0,0011	<0,0001	0,0014	0,0008	>0,9999	0,006
AZ 0.01% SF	15,12	1,213	AZ 0.01% 7D SF	5,476	0,7427	1,756	5,617	9,53	8,762	5,48	2,071		<0,0001	<0,0001	<0,0001	<0,0001	>0,9999	>0,9999
AZ 0.05% 0D	0,9844	0,3526	AZ 0.05% 0D	0,007	5,846	6,363	0,1138	0,5988	0,991	0,01012	4,408	5,466		>0,9999	>0,9999	>0,9999	<0,0001	<0,0001
AZ 0.05%7D	2,718	0,4856	AZ 0.05%7D	1,698	8,139	9,153	1,839	2,134	1,366	1,702	5,325	7,397	1,689		>0,9999	>0,9999	<0,0001	<0,0001
AZ 0.05% SF	3,882	0,4417	AZ 0.05% 7D SF	2,193	7,169	8,182	2,334	3,104	2,336	2,198	4,355	6,426	2,184	0,9703		>0,9999	<0,0001	<0,0001
MN 0D	0,98	0,352	MN 0D	0,038	5,905	6,423	0,06894	0,658	1,05	0,03479	4,468	5,526	0,04491	1,748	2,243		<0,0001	<0,0001
MN 7D	14,26	1,028	MN 7D	5,37	0,9495	1,963	5,511	9,324	8,556	5,374	1,865	0,2067	5,361	7,19	6,22	5,42		>0,9999
MN SF	18,89	0,7204	MN 7D SF	6,473	1,209	0,1957	6,614	11,48	10,71	6,477	4,023	1,952	6,463	9,348	8,378	6,523	2,159	

	SUC 0D	SUC 7D	SUC SF	MP 0D	MP 7D	MP SF	AZ 0.01% 0D	AZ 0.01% 7D	AZ 0.01% SF	AZ 0.05% 0D	AZ 0.05%7D	AZ 0.05% SF	MN 0D	MN 7D	MN SF
data group	a	b,c	b	a	a	a	a	c	b,c	a	a	a	a	b,c	b

Number of dead and alive insects in survival experiments of Figure 2.

SUC	2 days			males	dead	alive	total	SUC	7 days			males	dead	alive	total
females	dead	alive	total					females	dead	alive	total				
Rep.1	0	10	10	Rep.1	0	10	10	Rep.1	0	10	10	Rep.1	0	10	10
Rep.2	0	10	10	Rep.2	0	10	10	Rep.2	0	10	10	Rep.2	0	10	10
Rep.3	0	10	10	Rep.3	0	10	10	Rep.3	4	6	10	Rep.3	5	5	10
total	0	30	30	total	0	30	30	total	4	26	30	total	5	25	30

MP 0.25%	2 days			males	dead	alive	total	MP 0.25%	7 days			males	dead	alive	total
females	dead	alive	total					females	dead	alive	total				
Rep.1	0	10	10	Rep.1	0	10	10	Rep.1	1	9	10	Rep.1	0	10	10
Rep.2	0	10	10	Rep.2	0	10	10	Rep.2	4	6	10	Rep.2	3	7	10
Rep.3	0	10	10	Rep.3	0	10	10	Rep.3	5	5	10	Rep.3	5	5	10
total	0	30	30	total	0	30	30	total	10	20	30	total	8	22	30

AZ 0.01%	2 days			males	dead	alive	total	AZ 0.01%	7 days			males	dead	alive	total
females	dead	alive	total					females	dead	alive	total				
Rep.1	2	8	10	Rep.1	1	9	10	Rep.1	2	8	10	Rep.1	1	10	11
Rep.2	0	10	10	Rep.2	0	10	10	Rep.2	1	9	10	Rep.2	2	7	9
Rep.3	0	10	10	Rep.3	0	10	10	Rep.3	8	2	10	Rep.3	6	5	11
total	2	28	30	total	1	29	30	total	11	19	30	total	9	22	31

AZ 0.05%	2 days			males	dead	alive	total	AZ 0.05%	7 days			males	dead	alive	total
females	dead	alive	total					females	dead	alive	total				
Rep.1	0	10	10	Rep.1	0	10	10	Rep.1	3	7	10	Rep.1	9	1	10
Rep.2	0	10	10	Rep.2	0	10	10	Rep.2	3	7	10	Rep.2	2	8	10
Rep.3	0	10	10	Rep.3	0	10	10	Rep.3	6	4	10	Rep.3	6	4	10
total	0	30	30	total	0	30	30	total	12	18	30	total	17	13	30

Number of dead and live flies for survival data in Figure 2

MN 0.01% 2 days

females	dead	alive	total	males	dead	alive	total
Rep.1	3	7	10	Rep.1	0	10	10
Rep.2	0	10	10	Rep.2	0	10	10
Rep.3	6	4	10	Rep.3	10	0	10
total	9	21	30	total	10	20	30

MN 0.01% 7 days

females	dead	alive	total	males	dead	alive	total
Rep.1	3	7	10	Rep.1	0	10	10
Rep.2	4	6	10	Rep.2	3	7	10
Rep.3	9	1	10	Rep.3	10	0	10
total	16	14	30	total	13	17	30

MP + MN 2 days

females	dead	alive	total	males	dead	alive	total
Rep.1	1	9	10	Rep.1	2	8	10
Rep.2	0	10	10	Rep.2	0	10	10
Rep.3	3	7	10	Rep.3	0	10	10
Rep.4	4	6	10	Rep.4	5	5	10
Rep.5	1	9	10	Rep.5	1	9	10
total	9	41	50	total	8	42	50

MP + MN 7 days

females	dead	alive	total	males	dead	alive	total
Rep.1	10	0	10	Rep.1	7	3	10
Rep.2	0	10	10	Rep.2	0	10	10
Rep.3	10	0	10	Rep.3	5	5	10
Rep.4	10	0	10	Rep.4	10	0	10
Rep.5	6	4	10	Rep.5	2	8	10
total	36	14	50	total	24	26	50

Percentages of dead and live flies for survival data in Figure 2

SUC	2D	7D	MP	2D	7D	AZ 0.01%	2D	7D	AZ 0.05%	2D	7D	MN	2D	7D	MP+MN	2D	7D
alive	100	87	alive	100	67	alive	93	63	alive	100	60	alive	70	47	alive	82	28
dead	0	13	dead	0	33	dead	7	37	dead	0	40	dead	30	53	dead	18	72

SUC	2D	7D	MP	2D	7D	AZ 0.01%	2D	7D	AZ 0.05%	2D	7D	MN	2D	7D	MP+MN	2D	7D
alive	100	83	alive	100	73	alive	97	71	alive	100	43	alive	67	57	alive	84	52
dead	0	17	dead	0	27	dead	3	29	dead	0	57	dead	33	43	dead	16	48

Statistical analysis (contingency tables) of survival data in Figure 2C

Fisher (x SUC)

Females	MP	AZ 0.01%	AZ 0.05%	MN	MP+MN
2D	>0.9999	0,4915	>0.9999	0,0019	0,0229
7D	0,1253	0,0716	0,0391	0,0022	<0.0001

Males	MP	AZ 0.01%	AZ 0.05%	MN	MP+MN
2D	>0.9999	>0.9999	>0.9999	0,0008	0,0222
7D	0,5321	0,3627	0,0028	0,047	0,0076

Statistical analysis (Log-rank test) of survival data in Figures 2A and 2B

Log-rank

	SUC	MP	AZ 0.01%	AZ 0.05%	MN	MP+MN
Chi2 - p	a	a,b	b,c	b,c	c,d	d
Females	SUC	MP	AZ 0.01%	AZ 0.05%	MN	MP+MN
SUC		0,0694	0,0331	0,0206	0,0006	<0,0001
MP	3,298		0,6686	0,5952	0,0388	<0,0001
AZ 0.01%	4,541	0,1832		0,9443	0,1025	0,0024
AZ 0.05%	5,364	0,2823	0,00488		0,0964	0,0017
MN	11,92	4,268	2,666	2,764		0,3851
MP+MN	25,02	25,02	9,252	9,876	0,7545	

	SUC	MP	AZ 0.01%	AZ 0.05%	MN	MP+MN
Chi2 - p	a	a,b	a,c	c	b,c	c
Males	SUC	MP	AZ 0.01%	AZ 0.05%	MN	MP+MN
SUC		0,3512	0,2095	0,0014	0,0134	0,0036
MP	0,8691		0,723	0,0194	0,0797	0,0355
AZ 0.01%	1,575	0,1257		0,0555	0,1507	0,0825
AZ 0.05%	10,16	5,462	3,667		>0,9999	0,9195
MN	6,121	3,071	2,065	0		0,9614
MP+MN	8,476	4,423	3,015	0,01022	0,00235	

Tables for contingecy analysis (Log-rank test) of survival data in Figure 2.

Females

day	SUC	day	MP	day	AZ 0.01%	day	AZ 0.05%	day	MN	day	MP+MN
7	1	7	1	2	1	7	1	2	1	2	1
7	1	7	1	2	1	7	1	2	1	2	1
7	1	7	1	7	1	7	1	2	1	2	1
7	1	7	1	7	1	7	1	2	1	2	1
7	0	7	1	7	1	7	1	2	1	2	1
7	0	7	1	7	1	7	1	2	1	2	1
7	0	7	1	7	1	7	1	2	1	2	1
7	0	7	1	7	1	7	1	2	1	2	1
7	0	7	1	7	1	7	1	2	1	2	1
7	0	7	1	7	1	7	1	7	1	7	1
7	0	7	0	7	1	7	1	7	1	7	1
7	0	7	0	7	0	7	1	7	1	7	1
7	0	7	0	7	0	7	0	7	1	7	1
7	0	7	0	7	0	7	0	7	1	7	1
7	0	7	0	7	0	7	0	7	1	7	1
7	0	7	0	7	0	7	0	7	1	7	1
7	0	7	0	7	0	7	0	7	0	7	1
7	0	7	0	7	0	7	0	7	0	7	1
7	0	7	0	7	0	7	0	7	0	7	1
7	0	7	0	7	0	7	0	7	0	7	1
7	0	7	0	7	0	7	0	7	0	7	1
7	0	7	0	7	0	7	0	7	0	7	1
7	0	7	0	7	0	7	0	7	0	7	1
7	0	7	0	7	0	7	0	7	0	7	1
7	0	7	0	7	0	7	0	7	0	7	1
7	0	7	0	7	0	7	0	7	0	7	1
7	0	7	0	7	0	7	0	7	0	7	1
7	0	7	0	7	0	7	0	7	0	7	1
7	0	7	0	7	0	7	0	7	0	7	1
7	0	7	0	7	0	7	0	7	0	7	1
										7	1
										7	1
										7	1
										7	1
										7	1
										7	1
										7	0
										7	0
										7	0
										7	0
										7	0
										7	0
										7	0
										7	0
										7	0
										7	0
										7	0
										7	0
										7	0
										7	0

Tables for contingecy analysis (Log-rank test) of survival data in Figure 2.

Males

day	SUC	day	MP	day	AZ 0.01%	day	AZ 0.05%	day	MN	day	MP+MN
7	1	7	1	2	1	7	1	2	1	2	1
7	1	7	1	7	1	7	1	2	1	2	1
7	1	7	1	7	1	7	1	2	1	2	1
7	1	7	1	7	1	7	1	2	1	2	1
7	1	7	1	7	1	7	1	2	1	2	1
7	0	7	1	7	1	7	1	2	1	2	1
7	0	7	1	7	1	7	1	2	1	2	1
7	0	7	1	7	1	7	1	2	1	2	1
7	0	7	0	7	1	7	1	2	1	7	1
7	0	7	0	7	0	7	1	2	1	7	1
7	0	7	0	7	0	7	1	7	1	7	1
7	0	7	0	7	0	7	1	7	1	7	1
7	0	7	0	7	0	7	1	7	1	7	1
7	0	7	0	7	0	7	1	7	0	7	1
7	0	7	0	7	0	7	1	7	0	7	1
7	0	7	0	7	0	7	1	7	0	7	1
7	0	7	0	7	0	7	1	7	0	7	1
7	0	7	0	7	0	7	0	7	0	7	1
7	0	7	0	7	0	7	0	7	0	7	1
7	0	7	0	7	0	7	0	7	0	7	1
7	0	7	0	7	0	7	0	7	0	7	1
7	0	7	0	7	0	7	0	7	0	7	1
7	0	7	0	7	0	7	0	7	0	7	1
7	0	7	0	7	0	7	0	7	0	7	1
7	0	7	0	7	0	7	0	7	0	7	0
7	0	7	0	7	0	7	0	7	0	7	0
7	0	7	0	7	0	7	0	7	0	7	0
7	0	7	0	7	0	7	0	7	0	7	0
7	0	7	0	7	0	7	0	7	0	7	0
7	0	7	0	7	0	7	0	7	0	7	0
										7	0
										7	0
										7	0
										7	0
										7	0
										7	0
										7	0
										7	0
										7	0
										7	0
										7	0
										7	0
										7	0
										7	0
										7	0
										7	0
										7	0
										7	0
										7	0
										7	0

Standard curve mandelonitrile (Fig. 3B)

ng/μL	Area
0,75	3.670
1,5	9.815
3	22.104
15	120.420
30	243.315
60	489.105
R	1,000
Intercept	-2475
Slope	8193

Quantification of mandelonitrile in sugar baits before and after 7 days, with or without sandflies

	0 day		7 days	
	CTR	SF	CTR	SF
	3,298	2,762	0	0
	3,076	2,839	0	0
	2,100	1,175	0	0
	2,484	1,934	0	0
	5,753	6,468	0	0
	5,929	5,380	0	0
	6,146	5,671	0	0
	6,157	6,320	0	0
	21,909	29,350	0	1,37
	22,362	27,691	1,33	1,7
	25,308	28,966	0	1,49
	23,902	31,210	0	0
	36,213	26,510	0	0
	30,477	18,195	1,33	0
	25,262	22,219	0	0
	16,895	21,107	0	0
	0,000	0,439	0	0
	0,501	0,603	0	0
	0,370	0,499	0	0
	0,000	0,383	0	0
average	11,907	11,986	0,133	0,228
SD	11,911	11,933	0,409	0,559
SEM	2,663	2,668	0,092	0,125

Mann-Whitney test (0D x 7D)

	U	p
CTR	26	< 0.0001
SF	15	< 0.0001

Tables for contingecy analysis (Log-rank test) of survival data in Figure 4A.

days	CTR	days	CTR	days	MN	days	MN
24	1	18	1	3	1	18	1
31	1	21	1	22	1	21	1
43	1	23	1	27	1	23	1
43	1	23	1	27	1	23	1
48	1	25	1	31	1	23	1
48	1	28	1	36	1	25	1
48	1	30	1	38	1	25	1
50	1	37	1	43	1	32	1
52	1	42	1	43	1	35	1
52	1	44	1	43	1	39	1
55	1	49	1	55	1	46	1
59	1	51	1	57	1	46	1
59	1	61	1	59	1	7	1
66	1	16	1	66	1	18	1
66	1	21	1	71	1	21	1
69	1	21	1	71	1	25	1
71	1	25	1	71	1	35	1
71	1	25	1	71	1	35	1
71	1	28	1	73	1	37	1
71	1	32	1	83	1	46	1
71	1	32	1	97	1	46	1
73	1	35	1	108	1	49	1
76	1	39	1	111	1	51	1
76	1	46	1	122	1	51	1
129	1	49	1	132	1	53	1
7	1	49	1	11	1	53	1
11	1	49	1	14	1	56	1
11	1	51	1	14	1	60	1
14	1	56	1	14	1		

Oviposition in choice assay (Fig. 4B)

number of eggs in each food pot (CC2 and CC2+MN in the same cage)

	CC2	CC2+MN
Cage 1	31	0
Cage 2	1	0
Cage 3	9	0
Cage 4	0	0
Cage 5	154	0
Cage 6	0	0
Cage 7	11	0
Cage 8	80	0
Cage 9	44	0
Cage 10	145	0
Cage 11	30	0
Cage 12	176	0
Cage 13	112	0
Cage 14	148	0
Cage 15	84	0
Cage 16	103	0
Cage 17	19	0
Cage 18	0	0
Cage 19	22	0
Cage 20	0	0
Cage 21	15	0
Cage 22	94	0
Cage 23	17	0
Cage 24	19	0
average	55	0
SD	58	0
SEM	12	0

Survival of D. melanogaster exposed to sugar baits (Fig. 4C)

	Control (CC2)		live insects		
dias	replica 1	replica 2	replica 3	replica 4	replica 5
0	25	25	25	25	25
2	25	25	25	25	25
4	24	25	25	25	25
7	24	25	25	25	25
9	24	25	24	25	25
11	24	25	20	25	25
14	24	25	20	25	25

	CC2+MN		live insects		
dias	replica 1	replica 2	replica 3	replica 4	replica 5
0	25	25	25	25	25
2	25	24	25	24	25
4	25	24	24	24	25
7	25	24	24	24	24
9	25	24	23	24	21
11	24	23	21	22	21
14	24	21	20	20	21

Contingecy table for survival data (Fig 4C)

control

Rep 1		Rep 2		Rep 3		Rep 4		Rep 5	
days	event	days	event	days	event	days	event	days	event
4	1	14	0	9	1	14	0	14	0
14	0	14	0	11	1	14	0	14	0
14	0	14	0	11	1	14	0	14	0
14	0	14	0	11	1	14	0	14	0
14	0	14	0	11	1	14	0	14	0
14	0	14	0	14	0	14	0	14	0
14	0	14	0	14	0	14	0	14	0
14	0	14	0	14	0	14	0	14	0
14	0	14	0	14	0	14	0	14	0
14	0	14	0	14	0	14	0	14	0
14	0	14	0	14	0	14	0	14	0
14	0	14	0	14	0	14	0	14	0
14	0	14	0	14	0	14	0	14	0
14	0	14	0	14	0	14	0	14	0
14	0	14	0	14	0	14	0	14	0
14	0	14	0	14	0	14	0	14	0
14	0	14	0	14	0	14	0	14	0
14	0	14	0	14	0	14	0	14	0
14	0	14	0	14	0	14	0	14	0
14	0	14	0	14	0	14	0	14	0
14	0	14	0	14	0	14	0	14	0
14	0	14	0	14	0	14	0	14	0
14	0	14	0	14	0	14	0	14	0
14	0	14	0	14	0	14	0	14	0
14	0	14	0	14	0	14	0	14	0

Contingecy table for survival data (Fig 4C)

CC2+MN

Rep 1		Rep 2		Rep 3		Rep 4		Rep 5	
days	event	days	event	days	event	days	event	days	event
11	1	2	1	4	1	2	1	7	1
14	0	11	1	9	1	11	1	9	1
14	0	14	1	11	1	11	1	9	1
14	0	14	1	11	1	14	1	9	1
14	0	14	0	14	1	14	1	14	0
14	0	14	0	14	0	14	0	14	0
14	0	14	0	14	0	14	0	14	0
14	0	14	0	14	0	14	0	14	0
14	0	14	0	14	0	14	0	14	0
14	0	14	0	14	0	14	0	14	0
14	0	14	0	14	0	14	0	14	0
14	0	14	0	14	0	14	0	14	0
14	0	14	0	14	0	14	0	14	0
14	0	14	0	14	0	14	0	14	0
14	0	14	0	14	0	14	0	14	0
14	0	14	0	14	0	14	0	14	0
14	0	14	0	14	0	14	0	14	0
14	0	14	0	14	0	14	0	14	0
14	0	14	0	14	0	14	0	14	0
14	0	14	0	14	0	14	0	14	0
14	0	14	0	14	0	14	0	14	0
14	0	14	0	14	0	14	0	14	0
14	0	14	0	14	0	14	0	14	0
14	0	14	0	14	0	14	0	14	0
14	0	14	0	14	0	14	0	14	0

Oviposition during forced exposure (Fig. 4D)

Number of eggs layd per cage

	CC2					CC2+MN				
days	Replica 1	Replica 2	Replica 3	Replica 4	Replica 5	Replica 1	Replica 2	Replica 3	Replica 4	Replica 5
2	212	212	240	199	123	0	45	0	0	0
4	46	110	191	148	91	0	0	0	0	0
7	82	104	116	132	34	0	0	0	0	0
9	133	90	97	128	30	0	0	0	0	0
11	87	58	53	90	26	0	0	0	0	0
14	62	31	31	39	7	0	0	0	0	0

Mann-Whitney test, CC2 x CC2+MN

day	U	p
2	0	0,0079
4	0	0,0079
7	0	0,0079
9	0	0,0079
11	0	0,0079
14	0	0,0079
